# Progesterone-mediated effects on gene expression and oocyte-cumulus complex transport in the mouse fallopian tube

**DOI:** 10.1186/s12958-015-0038-8

**Published:** 2015-05-13

**Authors:** Anna Bylander, Lina Gunnarsson, Ruijin Shao, Håkan Billig, DG Joakim Larsson

**Affiliations:** Department of Infectious Diseases, Institute of Biomedicine, The Sahlgrenska Academy, University of Gothenburg, Guldhedsgatan 10, SE–413 46 Gothenburg, Sweden; Institute of Neuroscience and Physiology, the Sahlgrenska Academy, University of Gothenburg, Box 454, SE–405 30 Gothenburg, Sweden

**Keywords:** Fallopian tube, progesterone, gene expression, endothelin, gamete transport

## Abstract

**Background:**

The fallopian tube transports the gametes to the fertilization site and delivers the embryo to the uterus at the optimal time for implantation. Progesterone and the classical progesterone receptor are involved in regulating both tubal ciliary beating and muscular contractions, likely via both genomic and non-genomic actions.

**Methods:**

To provide more details of the underlying mechanisms, we investigated the effect of progesterone on gene expression in mice fallopian tubes *in vitro* at 20 min, 2 h and 8 h post progesterone treatment using microarray and/or quantitative PCR. In parallel, oocyte cumulus complex transport was investigated in ovulating mice that were injected with one of the progesterone receptor antagonists, Org 31710 or CDB2194.

**Results:**

Microarray analyses did not reveal any apparently regulated genes 20 min after progesterone treatment, consistent with the proposed non-genomic action of progesterone controlling ciliary beating. After 2 h, 11 genes were identified as up-regulated. Analyses using quantitative PCR at 2 h and 8 h showed a consistent up-regulation of endothelin1 and a down-regulation of its receptor Endothelin receptor A by progesterone. We also confirmed that treatment with progesterone receptor antagonists before ovulation accelerates the transport of the oocyte cumulus complex.

**Conclusions:**

This is the first study showing that progesterone regulates the expression of endothelin1 and endothelin receptor A in the fallopian tube. Together with previous studies of the effects of endothelin on muscular contractions in the fallopian tube, the results from this study suggest that endothelin is a mediator of the progesterone-controlled effects on muscular contraction and eventually gamete transport in the fallopian tube.

**Electronic supplementary material:**

The online version of this article (doi:10.1186/s12958-015-0038-8) contains supplementary material, which is available to authorized users.

## Background

The fundamental steps in female reproduction include the transport of the oocyte cumulus complex (OCC) from the site of ovulation to the site of fertilization and further transport of the fertilized embryo to the site of implantation. The fallopian tube provides the ways and means of this process by muscular contraction, ciliary movement and flow of secretions [1], which are influenced and affected by progesterone and the classical progesterone receptor (PGR) [2–4]. The roles of progesterone in the ovary, uterus and mammary gland have been well described, but its role and detailed mode of action in the fallopian tube are not as well defined. Overall, progesterone affects processes that influence and optimize the transport of the OCC to the site of fertilization as well as nourish and transport the embryo to the uterus. Because progesterone acts to increase the chance of a successful implantation, it is important to also reveal the mechanisms behind its actions in the fallopian tube [5–8].

Progesterone is involved in the regulation of the frequency of muscle contractions in human fallopian tubes *in vitro* in a dose-dependent manner [9]. The regulation of muscle contractions is complex and influenced by several factors, such as sex steroids, prostaglandins and endothelins [9–15]. Estrogen promotes muscular contraction, and progesterone promotes relaxation [10, 15]. Prostaglandins seem to have a dual effect on contractility where the E-series (PGE) relaxes muscle activity and the F-series (PGF) induces muscle activity. This response appears to be influenced by ovarian steroids, as progesterone increases the response to PGE1 and decreases the response to PGF2alpha [16]. Research have shown that the expression of PGE and PGF2alpha receptors are affected by mifepristone (RU486), an antagonist to both corticoid and progesterone receptors, suggesting that prostaglandin receptors in the fallopian tube are directly or indirectly regulated by progesterone [17]. The role of endothelins in the fallopian tube is relatively well established. Both endothelin 1 (EDN1) and endothelin 2 (EDN2) have been shown to increase muscular contractility in the fallopian tube [11–13]. One *in vitro* study *by Wanggren et al.* demonstrated the effects of 100 nM progesterone on muscle contractions in the fallopian tube within 20 min [9]. Such a rapid time course supports the hypothesis that progesterone regulates muscular activity through both transcriptional and non-transcriptional pathways. In the study by Wanggren, the effect of progesterone was not blocked by the antagonist mifepristone (RU486), which could indicate the involvement of a receptor other than the PGR. However, progesterone and RU486 was administered at the same time and at equal concentrations, which may not be sufficient for efficient blockage [18, 19].

The exact location of the PGR in the fallopian tube is under debate. Immunohistochemistry data suggest a location in the lower half of the cilia in both mouse and human fallopian tubes [20]. Another study suggests a PGR location in the nuclear compartments of the luminal, epithelial and smooth muscle cells in the mouse fallopian tube [21]. In addition to the PGR, there are several candidate receptors that could potentially mediate the non-transcriptional effects of progesterone, including the membrane progesterone receptors (mPRs) [22]. Two isoforms of mPRs are present in the fallopian tube in both humans and mice, and both isoforms are regulated by progesterone [23, 24].

It is now well documented that progesterone decreases the ciliary beat frequency (CBF) of the fallopian tube. In humans, a high concentration of progesterone (10 μmol/L) reduced the CBF by 40–50 % 24 h after exposure [2]. In a similar study on ciliary cells from bovine oviduct, 20 μmol/L progesterone caused a reduction in the CBF within 30 min after exposure [19]. Studies in our laboratory on ciliary cells from mice have demonstrated an even faster (10–20 min) significant reduction of CBF by progesterone, using physiologically relevant concentrations of progesterone in the range of 10–100 nmol/L [4, 18]. We have previously established the involvement of the classical PGR in these rapid effects of progesterone on CBF. The reduction in CBF was induced when ciliary cells were exposed both to progesterone and promegestone, a specific agonist to PGR, and the reduction in CBF was blocked when ciliary cells were pre-treated with excess RU486 before exposure to progesterone. In mice lacking a functional *Pgr,* there was no reduction in CBF after progesterone administration [18]. Taken together, these findings indicate that PGR mediate the rapid CBF response to progesterone in the fallopian tube. The rapid effects suggest a non-genomic response may be involved, but data on the potential rapid changes in gene transcription in the fallopian tube following progesterone treatment are not known.

Studies have also suggested that PGR directly affects OCC and embryo transport of the fallopian tube *in vivo*. In one study, rats were treated with RU486 from the day of ovulation after which the fallopian tubes and uteri were flushed at different time points. In rats treated with RU486, the eggs arrived approximately 11 h earlier to the uterus than in the control rats. In another study, mice were administered a daily subcutaneous injection of two different progesterone receptor antagonists, RU486 and ZK98734, or vehicle during the 3 first days of pregnancy, and the uteri were flushed on the afternoon of day 3. Both antagonists significantly increased the number of embryos in the uterus compared to the control i.e., stimulated premature entry to the uterus [25, 26].

We hypothesize that PGR-mediated gene expression in the fallopian tube is part of the short and/or long-term regulation of OCC transport. We have started to address this by investigating global gene expression in the mouse fallopian tube after progesterone treatment *in vitro* to exclude indirect effects mediated by modulating the secretion of other hormones within the hypothalamus-pituitary-gonadal axis. The timing of regulation as well as the identity of these genes could provide insight into the mechanisms of progesterone-regulated OCC transport*.* Furthermore, the functional role of progesterone and PGR in the transport of the OCC in the fallopian tube was confirmed. The distribution of the PGR in the mouse fallopian tube was also investigated.

## Methods

### Animals

All animal experiments were approved by the local animal ethics committee in Gothenburg, Sweden, (50/2011) to DGJL and (58/2007) to RS. The mice used in this study were all immature female (3.5–5 weeks old) C57BL/6 N mice from Charles River, Kisslegg, Germany. The animals were housed under a 12:12 h light-dark schedule at 21 °C ± 2 °C with unlimited access to food and water. Before the start of experiment, the animals were allowed to acclimate to the animal facilities for ≥ 5 days.

### *In vivo* treatment with progesterone antagonists and assessment of OCC transport

To investigate the role of PGR in OCC transport, ovulation was stimulated in 105 immature mice by intraperitoneal injection (i.p) of 5 IU Pregnant mare's serum gonadotropin (PMSG) (N .V. Organon, Oss, Holland) followed 48 h later by i.p injection of 5 IU of human chorionic gonadotropin (hCG) (N.V. Organon, Oss, Holland). In rodents, ovulation occurs 12–14 h after PMSG/hCG treatment. To determine whether a single dose of a PGR antagonist influences ovum transport, 40 of the PMSG/hCG-treated mice received an i.p. injection of Org31710 (8 μg/g) 6 h after the hCG injection, 25 were instead treated with another more specific PGR antagonist CDB2194 (8 μg/g) [27–29], whereas 40 control mice were administered human chorionic gonadotropin vehicle only. The time point for the antagonist injection was chosen because we wanted to administer the antagonist before the OCCs were released into the fallopian tube but not too early to risk blocking the ovulation process. Both antagonists were dissolved in ethanol and suspended in sesame oil, whereas control mice were administered ethanol and sesame oil only (i.p. injection volume 100 μl). Both Org31710 and CDB2194 are more potent and show less anti glucocorticoid activity than other PGR antagonists such as RU486 and ZK 98299 [30]. We use two antagonists to show with higher certainty that the effect is indeed through the PGR. The doses of Org31710 and CDB2194 were chosen based on previous studies [28, 30]. All drug solutions were freshly prepared before each experiment. Mice from each treatment group were killed by cervical dislocation, and their fallopian tubes were removed, dissected and examined under a dissecting microscope at 24, 27, 30, 36, and 48 h after hCG injection. The presence of OCCs in the fallopian tubes were determined by flushing the fallopian tubes and observing if any OCCs were retrieved under the microscope. Please note that the females were kept in cages without males, and thus, there were no fertilized eggs present.

### Tissue collection and progesterone exposure *in vitro*

Immature female mice (not treated with any hormones) were anesthetized by inhalation of isoflourane and then killed by inhalation of carbon dioxide followed by dissection. Both fallopian tubes were immediately removed and washed in pre-warmed (37 °C) G-MOPS™ (Vitrolife, Gothenburg, Sweden). G-MOPS is a medium specially developed for handling and manipulation of oocytes and embryos under normal air. After the removal of the broad ligaments, fat and blood vessels, the whole fallopian tubes, including all segments (total length approximately 18 mm) were longitudinally opened, cut into smaller pieces and then transferred to a new petri dish containing 2 ml pre-warmed G-MOPS™. In fallopian tubes from mice in this stage of development, ciliary cells and secretory cells are clearly visible [23]. Progesterone (>99 % purity, Sigma-Aldrich, St Louis, MO) was first dissolved in ethanol and then further dissolved in G-MOPS™. To add progesterone to the fallopian tubes, half of the medium in the petri dish was removed and replaced with an equal amount of medium containing progesterone and ethanol resulting in a final concentration of 100 nmol/L progesterone and 0.01 % (v/v) ethanol. The fallopian tubes in the control samples were exposed to ethanol (0.01 % (v/v)) only. The concentration of progesterone was based on results from our previous studies demonstrating clear effects on ciliary motility in this *in vitro* model [4, 18]. From each mouse, one fallopian tube was exposed to progesterone and one was used as the control. A total number of 48 mice and 96 fallopian tubes were used. The tissue samples were exposed for 20 min or 2 h, and immediately after exposure, they were snap-frozen in liquid nitrogen and stored at −80 °C until RNA extraction. To obtain sufficient quantities of RNA for the microarray analyses, six fallopian tubes (three animals) were pooled in one petri dish to form one sample. For each time point, we had four sample pairs resulting in a total of 16 samples. For additional gene expression analysis with quantitative PCR (qPCR), the fallopian tubes from an additional 36 mice were exposed to 100 nmol/L progesterone or control medium for 2 h or 8 h, resulting in four groups. For this analysis, less RNA was required, and the fallopian tube tissue from one mouse was sufficient to analyze as an independent sample. Thus, in total, nine samples from nine separate mice from each treatment group were analysed using qPCR. Immediately after exposure, the samples were snap−frozen in liquid nitrogen and stored at −80 °C.

### RNA extraction and gene expression analysis

Fallopian tube samples were removed from liquid nitrogen storage and homogenized using a Tissue Lyser (Qiagen, Hilden, Germany). Total RNA was extracted using the RNeasy Plus micro Kit (Qiagen) according to the manufacturer’s protocol. RNA quality and quantity were evaluated based on spectrophotometric measurements (Nanodrop 1000, NanoDrop Technologies, USA) and possible RNA degradation was measured with an RNA StdSens analysis kit and Experion (Experion™ Electrophoresis Station, Bio-Rad laboratories, Hercules, CA).

### Microarray analyses

The gene expression in the fallopian tube after exposure to progesterone for 20 min or 2 h was analysed by microarray performed at the Swegene Centre for Integrative Biology (SCIBLU) at Lunds University, Lund, Sweden. In short, 500 ng total RNA was processed using the Illumina®Totalprep-96RNA Amplification kit 4397949 RevD, (Illumina) according to the manufacturer’s instructions to generate double stranded cDNA. The cDNA was used as a template to produce biotin-labeled cRNA according to the manufacturer’s specifications. The cRNA was then hybridized on to MouseRef-8 v2 Expression BeadChip using Whole-Genome Gene Expression Direct Hybridization Assay (Illumina) following the manufacturer’s instructions. The Beadchips were scanned in the iScan system using the Standard Illumina iScan N240 scanning protocol. Experimental quality analyses, probe summarization and quantile normalization [31] were performed using the GenomeStudio software V2011.1. Probe sets without signal intensity, i.e., intensity above the median of negative control intensity signals, in 80 % of the samples were excluded. Probe sets with no gene annotations or expired annotations were filtered. A paired (four sample pairs exposed for 20 min and four sample pairs exposed for 2 h) Significance Analysis of Microarrays (SAM) [32] was preformed to identify differently expressed genes between the groups using the TMEV v4.0 software [33]. For each gene, a q-value was reported. The q-value is similar to the more well-known *p*-value but adapted to multiple testing situations. Genes with a q-value ≤20 were considered differentially expressed. Data from the microarray experiment is available with accession number GSE61407 at the NCBI Gene Expression Omnibus public repository (GEO) according to MIAME guidelines. The time point of 20 min was chosen to be able to identify possible genes involved in regulating the rapid reduction in CBF observed in our previous papers within that time period. We choose two hours as our second time point because we had previously established that the ciliary activity remains stable for at least two h of incubation [4, 18].

### Quantitative PCR

Four genes (*Edn1*, *Amigo2, Arlf4* and *Rasd1)* that tended to be differentially expressed after 2 h of progesterone exposure, as measured by the microarray analysis, were further assessed with quantitative PCR analysis. The genes were selected based on their fold change and a connection to progesterone or PGR in the literature. However, no PCR products were obtained for *Arlf4* (data not shown). The gene expression of five additional genes (*Pgr*, *Edn2*, *Edn3, Ednra, Ednrb)* was also assessed. These genes were not regulated according to the microarray but were selected based on a potential role in gamete transport. The analysis was performed on new samples from 36 mouse fallopian tubes exposed to progesterone (or control) for 2 h or 8 h *in vitro*. From each sample 1 μg of RNA was reverse-transcribed with a mixture of random hexamers and oligo (dt) primers, using the iScript cDNA Synthesis Kit (Bio-Rad). cDNA synthesis was performed according to the manufacturer’s protocol. The template cDNA, TaqMan Gene Expression master mix and pre-formulated primers and probes purchased as TaqMan Gene Expression assays (Life Technologies) were mixed and run in accordance with the manufacturer’s instructions. Taqman assay-identities are presented in Additional file 1: Table S1. Amplification reactions were carried out in triplicate with and started with a 10 min denaturation step at 95 °C followed by 40 cycles of 15 s at 95 °C and 1 minute at 60 °C. All amplification reactions were carried out in 385-well plates using the QuantStudio 12 K Flex (Applied Biosystems/Life Technologies; Stockholm, Sweden) and analysed with QuantStudio software. Technical replicates that evidently differed from the others were excluded from further analysis. The qPCR results were analysed by normalizing the median C_T_ value of the three technical replicates with the average of the median C_T_ values from two reference genes, and the resulting ΔC_T_ values were used for statistical analyses. *Rpl19* and *Hprt1* were used as housekeeping genes, both of which had stable mRNA expression levels in all samples and between groups (2 h *p* = 0.91, 8 h *p* = 0.55 and 2 h *p* = 0.91, 8 h *p* = 0.91, respectively) (two-sided *student’s t-test*).

### LacZ staining

To identify cells that express the PGR, we used fallopian tubes from PgrLacZ (+/−) mice. The LacZ mice were adult females who were kept without males, and we did not assess the stage of the cycle at the time of sampling. The fallopian tubes were dissected out and cleaned before they were frozen in OTC medium (Tissue-Tek O.T.C. Sakura Finetek Europe B.V). Then, they were cut into pieces (5–10 μM) in a cryostat (Leica CM3050S, Leica Microsystems AB) and placed on glass slides. To determine the β-galactosidase activity in the tissue we used a LacZ staining kit (InvivoGen, San Diego, USA). The glass slides were prepared according to the manufacturer’s protocol. In cells expressing the LacZ gene, β-galactosidase catalyzes the hydrolysis of X-Gal, producing a blue precipitate that can be visualized under a microscope.

### Statistical analyses

The statistical analysis was calculated based on the proportion of mice with at least one OCC present in the fallopian tube, not on the number of OCCs found in the fallopian tube. Fisher´s exact test were used to test if the ratios of mice with OCCs in the fallopian tube differed between mice given vehicle or either of the antagonists. Tests for differential gene expression (qPCR) between control and progesterone-exposed fallopian tubes were performed using two-sided student’s t-test with 0.05 as the alpha-value.

## Results

Table 1 presents the percent of mice with OCC present in the fallopian tube after treatment with two PGR antagonists or vehicle at different time points. In mice treated with Org 31710, a significant decrease in the ratio of mice with OCC present in the fallopian tube was found both at 30 h (*p* = 0.007) and 36 h (*p* = 0.0256) after hCG treatment. For mice treated with CDB2194, a similar significant decrease in the ratio of mice with OCC present was found at 27 h (*p* = 0.007) and 30 h (*p* = 0.0008) after hCG injection. At the doses given, there were no significant differences at any time point between the effects of the two antagonists. These results are consistent with the hypothesis that blockage of the PGR accelerates OCC transport.

**Table 1:** Progesterone receptor antagonists accelerate the transport of the oocyte cumulus complex (OCC) in mouse fallopian tube

	Percent of Mice with OCCs in the Fallopian Tube (number)				
	Hours after hCG Treatment				
Treatment	24	27	30	36	48
Vehicle	75 (8)	100 (8)	100 (8)	62.5 (8)	0 (8)
Org 31710 (8 μg/g)	100 (8)	62.5 (8)	25 (8)*	0 (8)*	0 (8)
CDB2194 (8 μg/g)	100 (5)	20 (5)*	0 (5)*	0 (5)	0 (5)

Total number of mice in each group is given in parenthesis. Vehicle or antagonist (Org 31710 or CDB2194) were administered 6 h after an injection with human chorionic gonadotropin (hCG). At each sampling point, Fisher’s exact test was used to calculate if the ratio of mice with OCC present in the fallopian tube differed between mice administered vehicle or either antagonist. The time points where the number of mice with OCC is significantly different from the vehicle group are indicated by an asterisk.

To identify possible progesterone-dependent genes involved in gamete transport and in rapid reduction of CBF, we performed a microarray analysis on fallopian tubes isolated from mice and exposed *in vitro* to 100 nmol/L progesterone for 20 min and 2 h. Exposure to progesterone for 20 min did not induce any significant change in gene expression in the mouse fallopian tube as assessed by the microarray analysis.

**Fig. 1:**
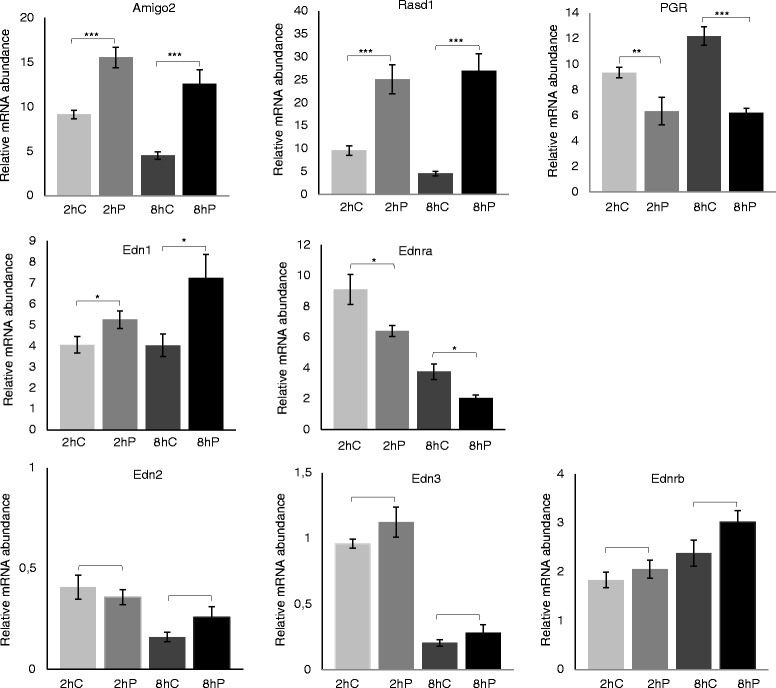
Progesterone-regulated gene expression in mouse fallopian tubes. Quantitative PCR analyses of 8 transcripts in mouse fallopian tubes treated with ethanol (EtOH) 0.01 % (v/v) as control (C) or progesterone (P; 100 nmol/L) in vitro for 2 h or 8 h. The relative mRNA abundance in the figure is presented as the normalized value (normalized to the housekeeping genes Hprt and Rpl19). Error bars represent standard error of mean. Significantly different expression between control and exposed fallopian tubes at the two time points is indicated by * (p < 0.05), ** (p < 0.01) or *** (p < 0.001)

Table 2 provides a list of 11 genes that were differently expressed in mouse fallopian tubes after exposure to progesterone for 2 h compared to control fallopian tubes. Nine of these genes (*Arlf4, Rasd1, Edn1, Irak2, Amigo2, Cxcl1, Edn1, P4ha2* and *Serpina3n*) produce a gene product that is involved in signalling processes. The progesterone induced gene expression changes of three of these genes (*Edn1, Amigo2* and *Rasd1*) were confirmed with qPCR analysis (Fig. 1). The mRNA abundance of *Amigo*2 was significantly higher after 2 and 8 h of exposure to progesterone (*p* <0.0001 for both time points, respectively) compared to the controls. The mRNA levels of *Rasd1* (*p* <0.0001 for both time points) and *Edn1* (*p* = 0.043 and *p* = 0.012) were also significantly increased at both time points. *EDN1* is known to be involved in muscular contraction of the human and bovine fallopian tube [12–14], and thus, the two other isoforms of endothelin (*Edn2* and *Edn3*) and its receptors (*Ednra and Ednrb*) were included in the analysis even though they were not identified as regulated by the microarray. We also included *Pgr* in the analysis because earlier studies have shown a regulation by progesterone in the fallopian tube [21]. *Ednra* (*p* = 0.01 and *p* = 0.004, 2 h and 8 h, respectively) and *Pgr* (*p* = 0.004 and *p* <0.0001) had a lower mRNA abundance after progesterone exposure, whereas there was no significant treatment effect for *Edn2* (*p* = 0.63 and 0.10) and *Edn3* (*p* = 0.25 and 0.29).

**Table 2:** Genes regulated by progesterone after 2 h in mouse fallopian tubes

Gene symbol	Gene name	Selected GO-terms (Biological Process)	Fold-change
*Serpina3n*	Serpin Peptidase Inhibitor, Clade A, member 3	Response to peptide hormone	1.4
*Edn1*	Endothelin-1	Intracellular signaling cascade, Maternal process involved in parturition, Positive regulation of smooth muscle contraction	1.3
*Rasd1*	RAS, dexamethasone-induced 1	Intracellular signaling cascade, nitric oxide mediated signal transduction, small GTPase mediated signal transduction	1.4
*Slc25a33*	Solute carrier family 25 member 33	Small molecule transport	1.6
*Tigd2*	Tigger transposable element-derived protein 2	DNA binding	1.9
*Arfl4*	ADP-ribosylation factor like 4D	Intracellular signaling cascade	1.9
*Irak2*	Interleukin-1 receptor associated kinase 2	Intracellular signaling cascade	1.3
*Cxcl1*	chemokine (C-X-C motif) ligand 1	Positive regulation of cytosolic calcium ion concentration	1.4
*Glrx*	Glutaredoxin	AtPase binding	1.9
*Amigo2*	Adhesion molecule with ig like domain 2	Cell adhesion, negative regulation of programmed cell death	1.3
*P4HA2*	Prolyl 4 hydroxylase alpha polypeptide II	Oxidation-reduction process	1.3

Mice fallopian tubes were treated in vitro with progesterone (100 nmol/L) or vehicle for 2 h and analysed by microarray. Eleven genes were differentially expressed (q-value ≤20 %). The fold-change refers to progesterone versus vehicle-treated fallopian tubes. A positive value indicates up-regulation by progesterone.

**Fig. 2:**
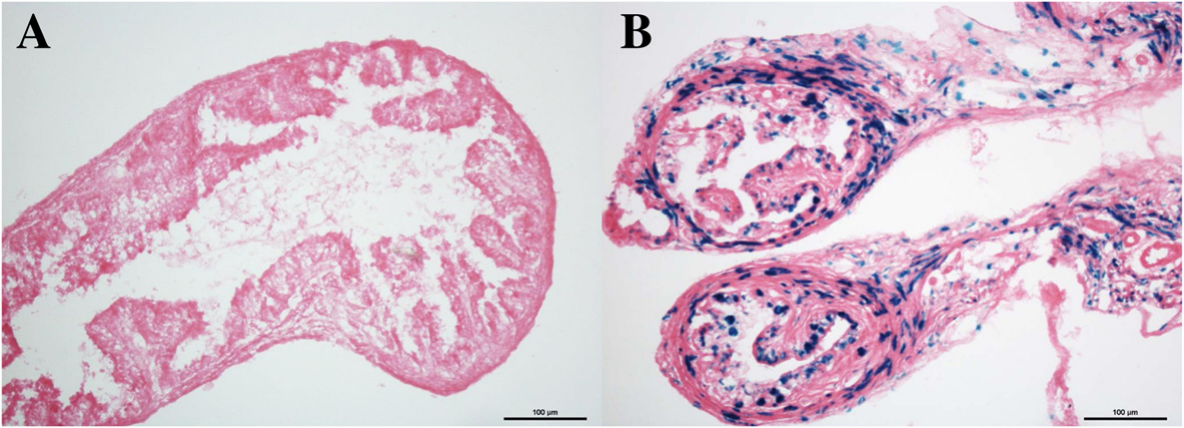
Localization of the PGR in mouse fallopian tubes. Fallopian tubes from A, cycling, wild-type mice, and B; PGRLacZ mice samples were stained to detect β-galactosidase activity and the location of PGR. Positive blue staining was observed in the epithelial and muscle cells of PGRLacZ (+/−) mice

To investigate the location of PGR in the mouse fallopian tube, we used fallopian tubes from PGRLacZ mice and a staining kit to detect β-galactosidase activity. As shown in Fig. 2, positive staining was observed in both epithelial and muscle cells.

## Discussion

Previous studies reporting the localization of PGR in the fallopian tube has been based on immunostaining [20, 21]. Here, we applied a method that does not depend on the specificity of antibodies. The results show that a sub-population of both epithelial cells and stromal cells express the PGR. This is consistent with the hypothesis that progesterone regulates both ciliary activity and muscle contractions via the PGR.

We also show that a single dose of two different PGR antagonists causes a rapid disappearance of the OCCs in the mouse fallopian tube. This is consistent with earlier studies showing that in rats and mice treated continuously with antagonists for PGR, the embryos arrived prematurely to the uterus [25, 26].

In a previous study from our laboratory, we showed that the rapid reduction of CBF, which was manifested within 20 min after progesterone administration, was inhibited if the ciliary cells were pre-treated with RU486 and in ciliary cells from mice lacking a functional *Pgr* [18]. This shows that PGR is most likely involved in this rapid regulation of the CBF. Combining these data with the effect of progesterone antagonists on OCC transport *in vivo* further indicates an important role of progesterone and the classical nuclear progesterone receptor in this process, which is at least partly mediated by changes in ciliary activity. In this study, we were not able to identify any changes in transcription abundance 20 min after progesterone treatment. Although it cannot be ruled out whether there are genes regulated after 20 min of progesterone exposure that were not discovered with the microarray analyses, our finding is consistent with the hypothesis that PGR regulates ciliary function in the fallopian tube via a non-genomic mechanism. Experiments with blockers of transcription or translation may provide more evidence for this hypothesis.

Transcriptional effects by progesterone were detectable after 2 h, as measured by microarray and qPCR. Nine of the genes that tended to be differentially expressed (*Arlf4, Rasd1, Edn1, Irak2, Amigo2, Cxcl1, Edn1, P4ha2* and *Serpina3n*) produce gene products that are involved in different signalling processes, and some of the genes are regulated by progesterone and *Pgr* in different cell lines in other studies [34–40]. *Amigo2* plays a role in cell-adhesion and cell migration. *Rasd1* encodes for a protein that is an activator of G-protein signalling. This gene is thought to be involved in nitric oxide signal transduction, which in the fallopian tube has been shown to affect muscle contractility [41, 42].

Most interestingly, *Edn1* was differentially expressed, both after 2 and 8 h of progesterone exposure. The absence of apparent regulation at 20 min suggests that progesterone-activation of the endothelin system via gene transcription is not a prerequisite for regulating ciliary function, but its regulation at later time points in the fallopian tube suggests that the endothelin system could be involved in other processes related to gamete transport. Indeed, EDN1 is known to induce muscle contractility in the fallopian tube [11, 12, 43]. Earlier studies show that estrogen and estrogen in combination with progesterone and luteinizing hormone significantly stimulates the production and secretion of EDN1 from bovine oviduct epithelial cells in culture. However, progesterone alone at concentrations ranging from 30 nmol/L to 3 μmol/L caused no significant increase in either production or secretion of EDN1 [44, 45]. In the present study progesterone induced the expression of the *Edn1* gene while causing a down-regulation of its receptor, *Ednra*. The reason for these somewhat contradictory results between the studies might be related to the fact that they are performed in different species using different methodologies. We measured the mRNA abundance in fallopian tubes from immature mice, after *in vitro* progesterone exposure. The other study measured the cell content and secretion of EDN1 from cultured oviductal epithelial cells from cows sampled during the estrous cycle. Consistent with our results, it has been previously demonstrated that *Edn1* is induced by Pgr-A in mouse granulosa cell culture [46].

EDN1 binds to two receptors EDNRA and EDNRB, with equal affinity, but the majority of its effect is mediated through EDNRA [47]. The localization of EDN1 within the human fallopian tube is suggested to be mainly within the tubal epithelium but also to a lesser extent in the muscle layer, while EDNRA is suggested to be dominant in the muscle layer [48]. The *Edn1, Edn2* and *Edn3* as well as the *Ednra* and *Ednrb* genes are reported to be expressed in bovine and mice oviducts during the estrous cycle [14, 49]. Consistently, we detected mRNA from all three endothelin isoforms and both receptors using qPCR. The expression of *Edn2, Edn3* and *Ednrb* was, however, very low compared to *Edn1* and *Ednra*, and only *Edn1* and *Ednra* were differently expressed after exposure to progesterone. In the bovine oviduct, the expression *of EDN1, EDNRA* and *EDNRB* mRNA was highest during the peri-ovulatory period. During this period, EDN1 also caused the highest increase in oviduct contraction. In bovine oviduct epithelial cells, EDN1 stimulates the production and release of prostaglandins. This suggests that in the bovine oviduct, EDN1 produced by the oviduct has a major role in the control of local contractions during the transport of gametes/embryos [14]. Our results indicate that the endothelin signalling is likely regulated, in turn, by progesterone.

We are not aware of any previous studies of *Ednra* regulation in the fallopian tube. Zhang et al. showed that *Ednra* was up-regulated in different tissues after progesterone treatment as well as in pregnant mice [46]. We found a down regulation of *Ednra* in the fallopian tube, suggesting that progesterone might regulate *Ednra*-expression in different directions depending on the tissue. When the controls for Ednra were compared, a decrease in mRNA abundance between the 2 h and 8 h samples was discovered. This decrease in abundance could either be due to less transcription and/or decreased stability of the mRNA.

In both the mouse and human fallopian tube, both isoforms of PGR are highly expressed [20]. In humans, the expression seems to vary across the menstrual cycle and differ between the different parts of the fallopian tube [50]. In our study, *Pgr mRNA* was down-regulated by progesterone, which is consistent with results from an earlier study showing that there was a time-dependent reduction of PGRA and PGRB protein expression in immature mice treated with a single dose of progesterone [21].

*Ednra* and *Pgr* were not markedly affected by the progesterone treatment as measured by the microarray analysis at 20 min or 2 h. However, with qPCR, significantly lowered mRNA levels after progesterone treatment were identified at both 2 h and 8 h in a separate experiment. One reason for this apparent difference at 2 h could be that both are receptors, and therefore, their expression is low compared to many other types of mRNA. A microarray has a considerably higher detection limit than a qPCR assay; thus, changes in genes that are hardly detectable by a microarray are much more subjected to noise than more highly expressed genes. A second reason might be that a gene might only be present and regulated in a small subset of the cells analysed, making it difficult to find small changes because they can drown in the noise from other cells. A third explanation could also be that the arrays were only performed with a replication of four per group, while the smaller amount of RNA required for the qPCR allowed us to analyze 9 biological replicates per treatment group, providing much higher statistical power.

A recent study investigated the differences in gene expression in fallopian tubes of mice completely lacking a functional *Pgr* (*Pgr*LacZ mice) and heterozygous mice (*Pgr*LacZ (+/−)) carrying one copy of the *Pgr* gene using both microarray and qPCR [51]. However, the overlap of the differentially expressed genes in that study and the genes found to be regulated directly by progesterone in the present study was small. Indeed, comparing the difference in gene expression between the two genotypes will reveal differences that are also indirectly affected by the lack of the receptor. Indeed, of the 9 genes investigated by qPCR after stimulation of ovulation in immature mice by hCG (thus inducing a progesterone-surge), 6 genes differed before treatment between the two genotypes. The authors identified an up-regulation of *Edn3* in heterozygous mice 8 hours after hCG treatment, while we did not observe any changes of *Edn3* either at 2 or 8 h after progesterone treatment. This suggests that the up-regulation of *Edn3* found during induced ovulation in the fallopian tube in the other study is not a direct effect of PGR activation.

## Conclusions

To our best knowledge, this is the first study demonstrating that *Edn1* and *Ednra* are directly regulated by progesterone in the mouse fallopian tube. We also confirm that PGR antagonists accelerate the transport of the OCC through the fallopian tube. Our results further support that progesterone and PGR plays an essential role in OCC transport that involves both non-genomic and genomic mechanisms. It is important to reveal the mechanisms and regulation of gamete transport in the fallopian tube because it is an essential step in natural reproduction and may contribute to a better understanding of the causes of infertility in women and the development of new and better contraceptives. To better understand the role of progesterone in the fallopian tube, studies on gene regulation in vivo would be important to perform in the future.

## Additional file

Additional file 1: Table S1. Taqman assay-id for primers and probes used for quantitative PCR analysis.

## Abbreviations

CBF: Ciliary beat frequency
EDN1: Endothelin 1
EDN2: Endothelin 2
EDN3: Endothelin 3
EDNRA: Endothelin receptor A
EDNRB: Endothelin receptor B
hCG: Human chorionic gonadotropin
mPRs: Membrane progesterone receptors
OCC: Oocyte cumulus complex
PGE: Prostaglandins E-series
PGF: Prostaglandins F-series
PGR: Progesterone receptor
PMSG: Pregnant mare's serum gonadotropin
qPCR: Quantitative PCR
RU486: Mifepristone
