# Supplementary material for: Progesterone-mediated effects on gene expression and oocyte-cumulus complex transport in the mouse fallopian tube
Source: Reprod Biol Endocrinol. 2015 May 13;13:40. doi: 10.1186/s12958-015-0038-8 (PMC4450483; doi:10.1186/s12958-015-0038-8)
Supplement: Additional file 1: Table S1. — Taqman assay-id for primers and probes used for quantitative PCR analysis. [file 12958_2015_38_MOESM1_ESM.pdf]

Supplemented table 1.

Gene name	**Taqman assay-id**
Amigo2	00662105_s1
Rasd1	00842185_g1
Arfl4	03031495_u1
Edn1	00438656_m1
Edn2	00432983_m1
Edn3	00432986_m1
EdnrA	01243722_m1
EdnrB	00432989_m1
Pgr	00435628_m1
Hprt	01545399_m1
Rpl19	02601633_g1

Taqman assay-id for primers and probes used for quantitative PCR analysis.
